# Phenotypic and Genotypic Detection of Extended-Spectrum Beta-Lactamase (ESBL) and Metallo-Beta-Lactamase (MBL) Genes in Multidrug-Resistant Acinetobacter baumannii Isolates Derived From Diabetic Foot Ulcer Patients

**DOI:** 10.7759/cureus.109329

**Published:** 2026-05-21

**Authors:** Hirdesh Kumari Gupta, Ramanath Karicheri, Abhishek Mehta

**Affiliations:** 1 Department of Microbiology, Index Medical College, Hospital, and Research Centre, Indore, IND; 2 Department of Microbiology, Government Medical College, Datia, IND

**Keywords:** acinetobacter baumannii, antimicrobial resistance, diabetic foot infection, extended spectrum beta lactamases (esbl), metallo-beta lactamases (mbl)

## Abstract

Objective: This study aimed to determine the antimicrobial resistance patterns of *Acinetobacter baumannii* isolates from diabetic foot infections. Secondary objectives were to identify phenotypic production of extended-spectrum beta-lactamases (ESBLs) and metallo-beta-lactamases (MBLs) and to detect associated resistance genes using polymerase chain reaction (PCR).

Methodology: This hospital-based cross-sectional study was conducted at a tertiary care center in Central India between June 2024 and September 2025. A total of 270 non-duplicate clinical samples from patients with diabetic foot ulcers were processed using standard microbiological techniques. Antimicrobial susceptibility testing was performed using the Kirby-Bauer disk diffusion method. Phenotypic detection of ESBL and MBL production was performed using disc-based methods, while genotypic detection of resistance genes was performed by PCR.

Results: Of the 270 samples analyzed, 227 (84.07%) showed A. baumanii bacterial growth. The majority of patients were male, with a mean age of 56 years. *A. baumannii *isolates exhibited high levels of resistance to beta-lactam antibiotics, fluoroquinolones, and carbapenems. Among the 50 isolates analyzed in detail, 21 (42%) were ESBL producers, 24 (48%) were MBL producers, and 8 (16%) demonstrated co-production of both enzymes. Genotypic analysis revealed the presence of multiple beta-lactamase resistance genes.

Conclusion: This study demonstrates a high prevalence of multidrug-resistant *A. baumannii* in diabetic foot infections, with frequent production of ESBL and MBL enzymes. These findings highlight the importance of continuous antimicrobial resistance surveillance and the judicious use of antibiotics to improve the management and clinical outcomes of diabetic foot infections.

## Introduction

Diabetes is a serious worldwide health issue. About 435 million people worldwide are currently affected. Experts believe that figure will grow to 728 million by 2045 [1]. One of the most serious problems associated with the condition of diabetes is diabetic foot infection (DFI). These infections are often caused by poor patient care and poor blood sugar control [2]. Failure to treat chronic wounds in diabetic patients may lead to these severe infections, such as cellulitis, abscesses, necrotizing fasciitis, gangrene, septic arthritis, and lower extremity osteomyelitis [3]. These complications are the driving force for global rates of disease, mortality, and health care costs [4].

Diabetic foot ulcers (DFUs), which precede many DFIs, have a lifetime prevalence of 15-25% among diabetic patients [5], and DFIs affect approximately 15% of this population [6]. These infections may be monomicrobial or polymicrobial. Among the causative pathogens, *Acinetobacter baumannii*, a Gram-negative coccobacillus known for its high pathogenicity, ranks third, after *Pseudomonas aeruginosa* and *Staphylococcus aureus*, as a leading cause of DFIs [7]. *A. baumannii *can cause numerous infections, including sepsis, urinary tract infections, and soft tissue infections. It is particularly a problem in people who are immunocompromised and in patients with cystic fibrosis [8]. Notably, infections caused by *A. baumannii *are linked to mortality rates of 11-33% in patients with DFIs [9].

A much-discussed issue with *A. baumannii *is its increasing antibiotic resistance. To a large extent, this issue is driven by mobile genetic factors associated with the horizontal transfer of resistance genes between and among species and generations [9]. Key enzymes, such as metallo-β-lactamases (MBLs) and extended-spectrum β-lactamases (ESBLs), confer resistance to multiple classes of antibiotics. Resistance genes, including blaVIM, blaIMP, blaTEM, and blaSHV, are particularly responsible for broad-spectrum cephalosporin resistance [10].

Despite the increasing global burden of antimicrobial resistance, there is a relative paucity of region-specific data on the resistance patterns and underlying molecular mechanisms of *A. baumannii *in DFIs, particularly in Central India. Variations in antimicrobial usage practices, healthcare infrastructure, and infection control measures may significantly influence local resistance trends, underscoring the need for context-specific surveillance data to inform clinical decision-making.

Given the clinical severity of DFIs and the therapeutic challenges posed by multidrug-resistant pathogens, systematic evaluation of both phenotypic resistance patterns and underlying genetic determinants is essential.

In this context, the present study was conducted with the primary objective of assessing the antimicrobial susceptibility patterns of *A. baumannii *isolated from DFIs in a tertiary care setting in Central India. The secondary objectives were to evaluate the phenotypic production of ESBLs and MBLs and to identify the associated resistance genes using genotypic methods. These findings aim to support evidence-based antimicrobial therapy and strengthen local infection control strategies.

## Materials and methods

Study design and setting

From June 2024 to September 2025, a cross-sectional study was conducted at the Department of Microbiology, Index Medical College and Hospital, Indore, Madhya Pradesh, India. The study was initiated after obtaining approval from the Institutional Ethics Committee (Approval No. MU/research/EC/PhD/2023/348).

Informed consent for participation and use of clinical samples was obtained from patients or waived by the Institutional Ethics Committee, as applicable, in accordance with institutional ethical guidelines. All laboratory procedures were performed in compliance with standard biosafety protocols and quality control guidelines.

Study population

The study population comprised patients with clinically diagnosed DFIs who attended the tertiary care hospital for inpatient or outpatient services during the study period. Clinical specimens were collected and processed in the microbiology laboratory for bacteriological analysis. Patient inclusion was based on diagnoses made by the treating surgeons or physicians [10].

Sample size determination

The sample size was calculated using the standard sample size formula used to estimate a single population proportion:

$[ n = \frac{Z^{2} \times p \times q}{d^{2}} ]$

Here, Z is the standard normal deviation for 95% confidence level (1.96); p is the expected proportion, q = 1 - p; d is the margin of error; and n is the required sample size. To ensure maximal precision, p was conservatively assumed to be 0.5, with a 6% margin of error (d = 0.06). This calculation yielded a minimum sample size of 267. To account for potential sample loss or processing issues, 270 non-duplicate clinical samples were included. Only one sample per patient was considered to prevent duplication.

Inclusion and exclusion criteria

Patients of either gender with a confirmed diagnosis of diabetes mellitus and clinical evidence of DFI were included. Eligible samples included aseptically collected pus, tissue, or exudate. For analysis, only the first isolate obtained from each patient during the study period was considered.

Wherever available, information regarding prior antibiotic exposure, including recent or short-term use for the current or other infections, was obtained from medical records to account for its potential influence on antimicrobial resistance patterns.

Exclusion criteria included patients without clinical signs of DFIs, duplicate or repeat samples, inadequately collected or improperly transported specimens, and patients receiving prolonged or undocumented antibiotic therapy before sample collection when reliable clinical details were unavailable.

Data collection procedure

Demographic and relevant clinical data, including age, gender, and clinical diagnosis, were obtained from laboratory requisition forms and hospital medical records. Wound samples were collected by trained healthcare personnel following standard aseptic precautions. The ulcer surface was cleaned and debrided to remove superficial contaminants before sample collection. Specimens were rapidly transferred to the microbiology lab for processing.

Isolation and Identification of Bacterial Isolates

Samples were inoculated into the respective culture media and incubated aerobically at 37 °C for 24-48 hours. The bacterial isolates were identified by Gram staining, colony morphology, and standard biochemical tests. *A. baumannii *was confirmed by standard microbiological methods, and the isolates were preserved at -80 °C in tryptic soy broth containing 15% glycerol for subsequent analysis.

Antimicrobial Susceptibility Testing

An antimicrobial susceptibility test (AST) was performed using the Kirby-Bauer disk diffusion technique on Mueller-Hinton agar. The procedure was based on the Clinical and Laboratory Standards Institute guidelines (CLSI M100, 2024) [11]. The tested antibiotics included imipenem, meropenem, ciprofloxacin, levofloxacin, piperacillin, piperacillin-tazobactam, gentamicin, tobramycin, tetracycline, ceftriaxone, ceftazidime, cefotaxime, cefepime, and ceftriaxone-sulbactam. Results were interpreted as susceptible, intermediate, or resistant according to CLSI breakpoints [11].

Phenotypic Detection of ESBL and MBL Production

The production of extended-spectrum beta-lactamase was screened using the double-disk synergy test. The results were then verified using the combined- disc method. The definition of ESBL production was "an increase of zone diameter of 5 mm or more, when using a cephalosporin disc with clavulanic acid, compared to the disc alone."

Production of MBLs was determined by the combined disk technique using imipenem and an imipenem-ethylenediaminetetraacetic acid (EDTA) disk. An increase in diameter of the zone of ≥ 7 mm, observed when the diameter of the zone appears on the disk of the culture medium with the solution of EDTA compared to the disk of culture medium with the solution of imipenem, indicates the production of MBL. Acinetobacter baumannii ATCC 19606 was used as the quality control strain [12].

Genomic DNA Extraction and PCR

Genomic DNA was extracted from phenotypically confirmed isolates using the Easy Tissue and Cell Genomic DNA Purification Kit (Qiagen, Germany) according to the manufacturer's instructions. DNA quality and concentration were assessed using spectrophotometry, and samples were stored at -20°C until further analysis.

Polymerase chain reaction (PCR) was performed to detect ESBL genes (blaTEM, blaSHV, blaCTX-M) and MBL genes (blaIMP, blaVIM, blaNDM-1) using previously published gene-specific primers. Each PCR reaction was carried out in a total volume of 25 µL containing template DNA, primers, master mix, and nuclease-free water [13].

Amplification was performed under the following cycling conditions: initial denaturation at 94°C for five minutes, followed by 30-35 cycles of denaturation at 94°C for 30 seconds, annealing at 52-60°C (depending on the primer set) for 30 seconds, and extension at 72°C for one minute, with a final extension at 72°C for seven minutes.

Amplified products were resolved by electrophoresis on 1.5% agarose gel, stained with ethidium bromide, and visualized under ultraviolet illumination. A 100 bp DNA ladder was used as a molecular size marker.

Data analysis 

Data were entered into Microsoft Excel (Microsoft® Corp., Redmond, WA) and cross-checked for accuracy and completeness. Descriptive statistical analysis was performed to summarize demographic characteristics, culture positivity rates, antimicrobial resistance patterns, and the distribution of ESBL and MBL producers.

Categorical variables were expressed as frequencies and percentages. Continuous variables, such as age, were presented as mean ± standard deviation. Data validation was performed through double-entry verification to minimize transcription errors.

## Results

A total of 270 clinical samples were collected from patients with DFUs during the study period. Of these, 227 (84.07%) samples yielded bacterial growth on culture. Most patients were middle-aged or elderly, with a mean age of approximately 56 years (Table 1).

**Table 1:** Demographic characteristics

Variables	Total, N (%)
Mean age (in years)	56 ± 8.4
Total sample collected	270 (100%)
Culture-positive samples	227 (84.07%
Culture-negative samples	43 (15.93%)

**Figure 1:**
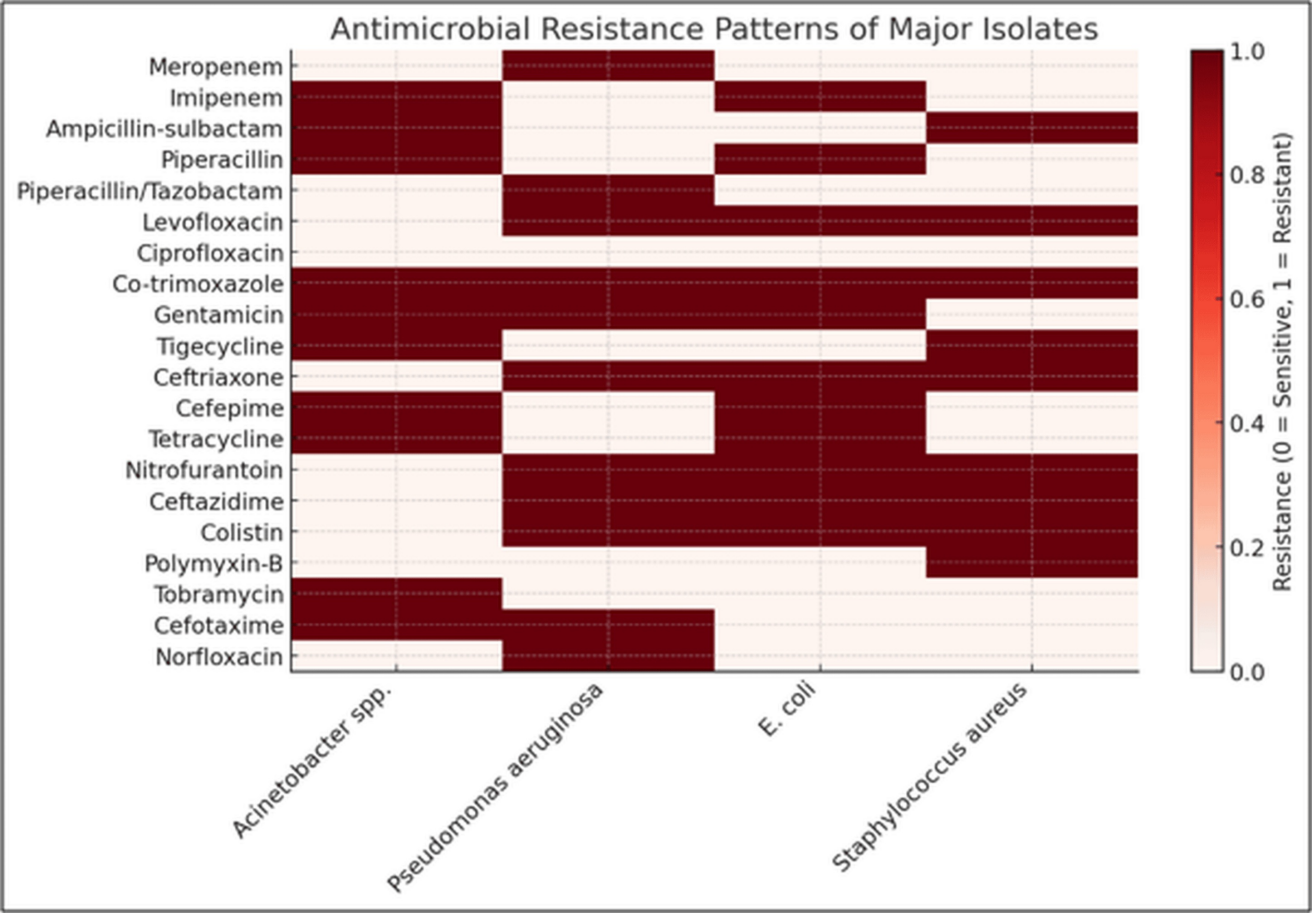
Antimicrobial resistance patterns

Figure 1 illustrates the antimicrobial resistance profile of *A. baumannii* isolates (n = 50) recovered from diabetic foot infections. A high level of resistance was observed to β-lactam antibiotics, with ceftazidime showing resistance in 50 (100%) isolates, followed by ceftriaxone and cefepime in 49 (98%) isolates each, and cefotaxime in 47 (94%) isolates. Among fluoroquinolones, ciprofloxacin and levofloxacin demonstrated resistance in 46 (92%) and 44 (88%) isolates, respectively. Carbapenem resistance was also notably high, with imipenem and meropenem showing resistance in 45 (90%) and 42 (84%) isolates, respectively. In contrast, comparatively lower resistance rates were observed for polymyxins, indicating that colistin and polymyxin B remain among the limited therapeutic options. Overall, the resistance pattern highlights the multidrug-resistant nature of *A. baumannii* isolates and the narrowing spectrum of effective antimicrobial agents.

Phenotypic analysis of 50 *A. baumannii *isolates revealed 21 (42%) ESBL-producing and 24 (48%) MBL-producing isolates. Co-production of both ESBL and MBL enzymes was observed in eight (16%) isolates. Additionally, 18 (36%) isolates did not demonstrate phenotypic production of either ESBL or MBL (Table 2).

**Table 2:** Phenotypic detection of ESBL and MBL among Acinetobacter baumannii isolates (n = 50) ESBL: extended-spectrum beta-lactamase; MBL: metallo-beta-lactamase

Phenotypic characteristic	Number of isolates (N)	Percentage (%)
ESBL producers	21	42
MBL producers	24	48
ESBL + MBL co-producers	8	16
Non-producers	18	36

Genotypic analysis revealed the presence of several ESBL and MBL genes among the isolates. The blaTEM gene was detected in 17 (34%) isolates, followed by blaCTX-M in 16 (32%) isolates and blaSHV in 12 (24%) isolates. Among MBL genes, blaNDM-1 was identified in 12 (24%) isolates, while blaVIM and blaIMP were detected in nine (18%) and seven (14%) isolates, respectively (Table 3).

**Table 3:** Distribution of ESBL and MBL genes among Acinetobacter baumannii isolates (n = 50)

Gene	Number of isolates (N)	Percentage (%)
blaTEM	17	34
blaCTX-M	16	32
blaSHV	12	24
blaNDM-1	12	24
blaVIM	9	18
blaIMP	7	14

## Discussion

Among 270 patients with DFUs, bacterial growth was detected in 227 patients, yielding a culture-positive rate of 84.07%. Males accounted for 65.55% of the study population, reflecting a male predominance consistent with previous Indian studies [13-15]. The average age of the patients was approximately 56 years, with most participants middle-aged or older, consistent with the demographic trends reported by Jassim et al. [16].

*A. baumannii* isolates exhibited high levels of antimicrobial resistance across multiple drug classes. Among β-lactam antibiotics, ceftazidime showed complete resistance (100%), followed by ceftriaxone and cefepime (98% each) and cefotaxime (94%). Fluoroquinolones also demonstrated significant resistance, with ciprofloxacin and levofloxacin exhibiting resistance rates of 92% and 88%, respectively. Carbapenem resistance was notably high, with imipenem and meropenem showing resistance rates of 90% and 84%. These findings underscore the multidrug-resistant nature of *A. baumannii* and align with international reports indicating increasing resistance trends in this pathogen [7-9,12,17].

Phenotypic analysis of the 50 *A. baumannii* isolates revealed that 21 (42%) were ESBL producers, while 24 (48%) were MBL producers. Co-production of ESBL and MBL enzymes was observed in 16% of isolates, posing significant therapeutic challenges due to limited treatment options. Previous studies have reported similar co-production rates, highlighting the growing clinical risk posed by β-lactamase-producing *A. baumannii* [18-20].

Genotypic analysis revealed resistance determinants in the isolates. The blaTEM gene was present in 34% of isolates, followed by blaCTX-M (32%) and blaSHV (24%). Among MBL genes, blaNDM-1 was the most prevalent (24%), followed by blaVIM (18%) and blaIMP (14%). These genes are known worldwide to be prevalent in multidrug-resistant *A. baumannii*. These are associated with resistance to carbapenems and extended-spectrum cephalosporins [18-20]. This study found high resistance rates in *A. baumannii*, which are in line with the World Health Organization's classification of the bacterium as a critical priority pathogen [8]. Ranjbar et al. reported fluoroquinolone resistance exceeding 80% in wound and burn isolates [21], comparable to the high ciprofloxacin and levofloxacin resistance observed here.

Polymyxin B and colistin are active for upwards of 70% of the isolates. However, the development of resistance to these last-line antibiotics is a cause for concern. Polymyxins are generally considered the final therapeutic option for multidrug-resistant *Acinetobacter*, and rising resistance further limits treatment choices. Additionally, a subset of isolates exhibited phenotypic-genotypic discordance, in which resistance genes were present but not phenotypically expressed, or vice versa. Similar results were obtained by Husna et al. [22], who attributed these discrepancies to factors such as gene regulation, low-level expression, or silent resistance genes. These results emphasize the importance of combining phenotypic and genotypic methods for accurate identification of resistance mechanisms.

Strength and limitations

This study has several notable strengths. It addresses a clinically significant and timely issue, antimicrobial resistance in *A. baumannii *isolated from diabetic foot infections, which remains a major global health concern. The use of both phenotypic and genotypic methods for the detection of ESBLs and MBLs enhances the reliability and scientific validity of the findings. Additionally, the inclusion of 270 non-duplicate clinical samples provides a reasonably adequate sample size for a single-center cross-sectional study.

However, the study also has important limitations. First, the cross-sectional design limits the ability to establish temporal relationships and may introduce selection bias. Second, as the study was conducted at a single tertiary care center, the findings may not be generalizable to other healthcare settings or geographic regions.

Although 270 samples were processed, only a subset of *A. baumannii *isolates underwent molecular analysis, potentially limiting the representation of circulating strains' genetic diversity. Furthermore, antimicrobial susceptibility testing was performed solely by disk diffusion, without determination of minimum inhibitory concentrations (MICs), which may affect the precision of resistance assessment.

The molecular analysis was limited to PCR-based detection of selected ESBL and MBL genes. While this approach identifies commonly prevalent resistance determinants, it does not provide a comprehensive characterization of resistance mechanisms. Advanced molecular techniques, such as gene sequencing and detailed genomic analysis, were not performed and could have provided deeper insights into the evolution and diversity of antimicrobial resistance.

In addition, the statistical analysis was limited to descriptive methods, without inferential analysis to assess associations or predictors. The study also lacked clinical outcome data, preventing correlation between antimicrobial resistance patterns and patient prognosis or treatment response. Finally, other resistance mechanisms, such as efflux pumps, alterations in porins, and additional β-lactamase variants, were not investigated.

## Conclusions

This study demonstrated that a high proportion of DFU samples were culture-positive, with *A. baumannii *isolates exhibiting marked resistance to β-lactam antibiotics, fluoroquinolones, and carbapenems. Among the analyzed isolates, 42% were ESBL producers, 48% were MBL producers, and 16% co-produced both enzymes, highlighting the significant burden of β-lactamase-mediated resistance. Genotypic analysis further confirmed the presence of multiple resistance determinants, with blaTEM, blaCTX-M, and blaNDM-1 being the most prevalent genes. These findings indicate a substantial circulation of multidrug-resistant *A. baumannii* strains harboring diverse β-lactamase genes in diabetic foot infections. The combined phenotypic and genotypic approach used in this study underscores the complexity of resistance mechanisms and supports the need for targeted antimicrobial strategies based on local resistance patterns.
